# Psyllium Fibre Inclusion in Gluten-Free Buckwheat Dough Improves Dough Structure and Lowers Glycaemic Index of the Resulting Bread

**DOI:** 10.3390/foods13050767

**Published:** 2024-03-01

**Authors:** Zihan Gao, Guangzhen Wang, Jing Zhang, Lichun Guo, Wei Zhao

**Affiliations:** 1 State Key Laboratory of Food Science and Technology, Jiangnan University, Wuxi 214122, China; 2 School of Food Science and Technology, Jiangnan University, Wuxi 214122, China

**Keywords:** psyllium fibre, gluten-free dough, dough structure, bread quality, starch digestibility

## Abstract

The demand for gluten-free (GF) bread is steadily increasing. However, the production of GF bread with improved baking quality and enhanced nutritional properties remains a challenge. In this study, we investigated the effects of adding psyllium fibre (PSY) in varying proportions to buckwheat flour on the dough characteristics, bread quality, and starch digestion properties of GF bread. Our results demonstrate that incorporating PSY contributes to the formation of a gluten-like network structure in the dough, leading to an increase in the gas holding capacity from 83.67% to 98.50%. The addition of PSY significantly increased the specific volume of the bread from 1.17 mL/g to 3.16 mL/g. Bread containing PSY displayed superior textural characteristics and colour. Our study also revealed that the inclusion of PSY reduced the digestibility of starch in GF bread. These findings highlight the positive impact of incorporating PSY into GF bread, suggesting its potential in guiding the production of GF bread with a lower glycaemic index. This may be particularly beneficial for individuals seeking to regulate their blood sugar levels or adopt a low-glycaemic diet.

## 1. Introduction

In recent years, there has been a significant and continuous increase in the demand for and consumption of gluten-free (GF) products. This growth can be primarily attributed to the rising prevalence of gluten-related disorders and the preference of some individuals for GF products [1]. According to a recent report, the global market size for GF products is projected to reach a value of 13.67 billion US dollars by 2030 [2]. Among the various GF products available, GF bread holds a prominent position and has the largest market share [3]. However, it is important to note that commercial GF bread often exhibits a high glycaemic index and unsatisfactory sensory qualities [1,4].

The production of GF bread typically involves the use of refined rice flour and purified potato starch, corn starch, or tapioca starch. Unfortunately, the reliance on these ingredients often results in GF bread with a low dietary fibre content and contributes to its high glycaemic index. This can give rise to various health-related concerns such as obesity, type 2 diabetes, and cardiovascular disease [5,6].

In terms of the baking quality of GF bread, there are several notable shortcomings. These include a reduced specific volume and increased hardness, which can be attributed to the absence of gluten [7]. In comparison to dough made with wheat, GF dough possesses a batter-like consistency and has a limited ability to retain gas [8]. Additionally, the batter-like nature of GF dough presents challenges for industrial production, particularly in terms of shaping. As a result, this limits the processability and variety of GF bread products [9].

There is a growing trend of incorporating pseudocereals as substitutes for traditional GF ingredients like potato starch or corn starch. These pseudocereals, such as buckwheat, offer higher nutritional value and have shown great potential in the production of GF bread. Buckwheat, in particular, is known for its abundance of resistant starch and its beneficial effects on blood glucose and lipid regulation, as well as improvements in gut flora [10,11,12]. The addition of various dietary fibres can have a significant impact on the texture, colour, moisture content, shelf life, and nutritional properties of GF bread [13]. Studies have shown that incorporating corn fibre, barley fibre, and rice bran into GF bread results in a more intense coloration, which is considered a desirable attribute for starch-based GF products [14,15]. Furthermore, the inclusion of dietary fibre effectively reduces bread staling, leading to a significant extension in its shelf life [16,17].

The type and particle size of the dietary fibre can affect the texture properties of GF bread. Generally, soluble fibre with smaller particle sizes can increase the specific volume and reduce the hardness of GF bread [18,19,20]. One particular soluble dietary fibre worth mentioning is psyllium fibre (PSY), which is derived from the husk of psyllium seeds. The consumption of PSY offers numerous physiological benefits, including blood sugar control, cholesterol reduction, the prevention of constipation, and a lowered risk of cardiovascular disease [21,22]. PSY has been shown to improve the batter-like quality of GF dough even at low levels, thanks to its remarkable water-holding and gelling properties [23,24]. However, the specific mechanism of how PSY regulates dough properties and improves the quality of GF bread remains unclear, and the effect of PSY on starch digestion in GF bread has yet to be revealed.

In this study, GF bread was prepared using buckwheat and PSY as the primary ingredient. The rheological behaviourand microstructural characteristics of the dough were investigated to clarify the effect of PSY on the dough properties. The quality of the GF bread was assessed in terms of specific volume, colour, structural characteristics, and texture. Additionally, the effect of the addition of PSY on starch digestibility was elucidated by analysing the changes to the viscosity of digesta and the kinetics of the enzymatic hydrolysis of starch in GF bread.

## 2. Materials and Methods

### 2.1. Materials and Chemical Reagents

Buckwheat flour was obtained from Liangchen Century Grain Co., Ltd. (Wuxi, China). Psyllium fibre was provided by Xuzhou Nature Food Co., Ltd. (Xuzhou, China). Instant dry yeast was purchased from Angel Yeast Co., Ltd. (Yichang, China). Maize oil and salt were obtained from local stores in Wuxi, China. Pepsin (P7000) and pancreatin (P7545) used for starch digestion assays were obtained from Sigma–Aldrich Chemical Co., Ltd. (St. Louis, MO, USA). Amyloglucosidase was obtained from Megazyme Inc. (Bray, Ireland). The glucose quantification kit, which employs the glucose oxidase method, was obtained from Nanjing JianCheng Bioengineering Institute (Nanjing, China). 

### 2.2. Preparation of Dough

Instant dry yeast, maize oil, and salt were combined at a proportion of 2%, 5%, and 1%, respectively, based on the total weight of buckwheat flour and PSY. When the amount of PSY was varied (0, 5, 10, 15, and 20%, *w*/*w*), buckwheat flour was added to maintain a constant total fibre concentration. The proportion of water (56%) was determined with a Mixolab instrument (Chopin Technologies, Paris, France). According to a faster Mixolab mixing protocol, the raw materials were then mixed in a bread blender for 8 min (4 min slow with 60 rpm/min, 4 min fast with 200 rpm/min). After mixing, a sample (500 g) of the dough was proved at 37 °C for 45 min at a relative humidity of 85%.

### 2.3. Analysis of the Rheological Properties of Dough

The dynamic rheological properties of the dough were measured using an MCR302 dynamic shear rheometer (Anton Paar Co., Graz, Austria) according to a previous study [25]. The linear viscoelastic region was determined by strain scan measurements. The frequency sweep measurements were performed at a frequency range from 0.1 to 100 Hz at a constant shear strain of 0.05%. The storage modulus (G′) and loss modulus (G″) were determined to assess the viscoelastic properties of the dough.

### 2.4. Analysis of Rheo-Fermentation Properties of Dough

A sample of 300 g of dough was proved at 37 °C for 3 h. The fermentation characteristics of the dough were determined using a Chopin F4 Rheofermentometer (Chopin Technologies, Paris, France) according to the manufacturer’s instructions. The temperature of the first plateau, which had a duration of 8 min, was 30 °C. The temperature was then increased with a gradient of 4 °C/min to the second plateau at 90 °C. The duration of the second plateau was 7 min. The temperature was then lowered with a gradient of 4 °C/min to the third plateau at 50 °C, and the duration of the third plateau was 5 min. During the proving process, the variations in the height of the dough, the total CO_2_ volume, and the CO_2_ retention rate were recorded.

### 2.5. Scanning Electron Microscopy 

Following fermentation, a small piece of the dough was immediately fixed by immersion in a 2.5% glutaraldehyde solution, and the dough was then dehydrated with an ethanol gradient. After freeze-drying for 48h, the dough was fixed on an aluminium holder using conductive adhesive and coated with a thin layer of gold. The microstructure of the dough was observed using an SU8100 cold field emission scanning electron microscope (Hitachi, Ltd., Tokyo, Japan) at an acceleration voltage of 3 kV and a magnification of 1000×. 

### 2.6. Preparation of Bread

The GF doughs were baked at 170 °C for 45 min in an SK2-621 oven (Sinmag Equipment Co., Ltd., Wuxi, China). After baking, the GF bread was cooled to ambient temperature and sealed in a plastic container and stored for less than 2 d before further analyses.

### 2.7. Determination of the Specific Volume and Baking Loss of Bread

The specific volume of GF bread was evaluated with the rapeseed displacement method, using changes to the weight and volume of the bread. The baking loss represents the loss of moisture from the bread during baking and was measured according to a previous study [15]. Equation (1) was used to determine baking loss.

(1) $$\mathrm{Baking}\;\mathrm{loss}\;\left(\%\right)=\frac{W_{1}-W_{2}}{W_{1}}\times 100\%$$

In this equation, *W*_1_ represents the weight of the dough before baking, and *W*_2_ represents the weight of the dough after baking.

### 2.8. Colour Measurement of Bread

The crust and crumb of GF bread were minced into particles of a uniform size, and the colour of these particles was measured using a colourimeter (CR-400, Konica Minolta Sensing, Inc., Osaka, Japan). The perceptual lightness (L*), the relative position of the shade between red and green (a*), and the relative position of the shade between blue and yellow (b*) were measured.

### 2.9. Textural Properties of Bread Crumbs

Bread crumb samples were obtained from the central portion of the bread and were cut into cubes measuring 2 × 2 × 2 cm^3^. The textural properties of these cubes, including hardness, springiness, and cohesiveness, were then analysed using a texture analyser (TA.XT. Plus, Stable Micro System, Surrey, UK) equipped with a P35 probe according to a published protocol [26]. The determination parameters were set as follows: a trigger force of 5 g, an interval time of 5 s between two compression cycles, and a strain of 50%.

### 2.10. In Vitro Analysis of the Starch Digestibility of Bread

The in vitro digestion characteristics of GF bread were investigated following the standard static method [27] with minor modifications. A minced crumb containing 0.5 g starch was mixed with deionized water, and the pH of the mixture was adjusted to 3.0 using 1.0 M HCl prior to pepsin digestion. Then, a portion (10 mL) of a simulated gastric fluid containing 4000 U/mL pepsin was added to the mixture. The reaction flask was sealed and incubated in a 37 °C water bath for 2 h with shaking at 150 rpm. The gastric digestion process was stopped by adjusting the pH of the mixture to 7.0, and then a portion (20 mL) of simulated intestinal fluid containing pancreatin was added to obtain a simulated intestinal mixture containing 200 U/mL of α-amylase. 

At predetermined time intervals, samples were collected and incubated in boiled water for 5 min to inactivate enzymes. Then, the samples were centrifuged at 10,000 rpm for 5 min to separate the undigested starch and starch hydrolysate. A solution containing amyloglucosidase was added to the supernatant to fully convert the starch hydrolysates into glucose [28]. The content of glucose was determined with a glucose oxidase-peroxidase kit. The starch hydrolysis rate and the ratio of rapidly digestible starch (RDS), slowly digestible starch (SDS), and resistant starch (RS) were calculated with Equation (2), Equation (3), Equation (4), and Equation (5), respectively.

(2) $$\mathrm{Hydrolysis}\;\mathrm{rate}\left(\%\right)=G_{t}\times 0.9/\mathrm{TS}\times 100\%$$

(3) $$RDS\left(\%\right)=\left(G_{20}-G_{0}\right)\times 0.9/\mathrm{TS}\times 100\%$$

(4) $$SDS\left(\%\right)=\left(G_{120}-G_{20}\right)\times 0.9/\mathrm{TS}\times 100\%$$

(5) $$RS\left(\%\right)=\left(\mathrm{TS}-\mathrm{RDS}-\mathrm{SDS}\right)\times 0.9/\mathrm{TS}\times 100\%$$

In these equations, *G*_t_ is the amount of glucose produced at predetermined time intervals; *G*_0_, *G*_20_, and *G*_120_ represent the amount of glucose produced at 0, 20, and 120 min from the start of starch digestion; and TS is the amount of total starch. The conversion factor of 0.9 was used to convert the glucose content into the corresponding amount of starch.

The starch digestion kinetics of GF bread were fitted with the nonlinear first-order rate equation as shown in Equation (6) [29].

(6) $$C_{t}=C_{\infty }\times \left(1-e^{-\mathrm{kt}}\right)$$

In this equation, *C_t_* is the amount of starch digested at digestion time *t* (min), *C_∞_* is the equilibrium amount of starch digested, and *k* (min^−1^) is the first-order kinetic coefficient.

### 2.11. Rheological Measurements of Bread Digesta

Samples were collected at 0, 20, and 120 min following the establishment of the intestinal phase as described in Section 2.10. The viscosity values of the digesta were measured using a dynamic shear rheometer (MCR302, Anton Paar Co., Ltd., Graz, Austria), equipped with a parallel plate apparatus (50 mm diameter, 1 mm gap). The test temperature was 37 °C, and the shear rates of steady shear measurements ranged from 1 to 100 s^−1^ [30].

### 2.12. Statistical Analysis

All experiments were performed in triplicate. The results are presented as the mean ± standard deviation (SD). Analysis of variance (ANOVA) analyses were conducted using the SPSS 19.0 software (SPSS Software Inc., Chicago, IL, USA). For statistical analysis, the Duncan test was employed, with significance differences considered at the *p* < 0.05 level. All figures were made with GraphPad Prism 9 (GraphPad Software Inc., San Diego, CA, USA). 

## 3. Results and Discussion

### 3.1. Rheological Properties of Dough

The equilibrium between dough elasticity and viscosity influences gas preservation and the development of a porous structure during proofing and baking [17,31]. The qualities of the dietary fibre in the mixture can influence the rheological properties of dough, thereby impacting the structure and texture of the bread [32]. 

**Figure 1:**
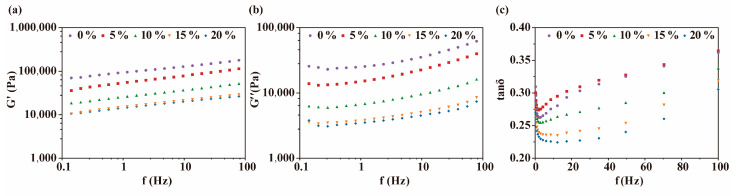
The dependence of rheological properties of dough on the proportion of PSY. (**a**) The effects of the concentration of PSY (0, 5, 10, 15, and 20%) on the storage modulus (G′). (**b**) The effects of PSY on the loss modulus (G″). (**c**) The effects of PSY on the phase shift tangent (tanδ) value.

The value of G′ is commonly used to indicate the elasticity and rigidity of dough [33]. Here, a control dough without the addition of PSY showed the highest value of G′, indicating its high resistance to deformation (Figure 1a). Compared with the control dough, the value of G′ decreased with the addition of PSY. This reduction in G′ suggests a softer texture of the dough and an enhanced capacity for expansion during fermentation. The decrease in dough rigidity can be attributed to the increased water content and the envelopment of starch particles by PSY, which effectively lubricated the dough [34,35]. This finding is consistent with previous studies that demonstrated how the addition of inulin to GF dough reduced the rigidity of the dough [18]. The value of G″ represents the viscosity of the dough. The addition of PSY decreased the value of G″ (Figure 1b); low viscosity generally promotes the expansion of gas cells.

The tanδ value, which represents the ratio of G″ to G′, is utilised as a gauge of the viscoelastic properties of dough [36,37]. Interestingly, upon the addition of more than 5% PSY, a notable decrease in the tanδ value was observed. This decline indicates that the inclusion of PSY enhances the contribution of elastic components to the viscoelastic behaviour of GF dough, rendering it more akin to a gel-like material (Figure 1c). This effect of PSY on lowering the viscoelastic property of dough may be due to the ability of PSY to reduce the water content in the dough.

### 3.2. Rheo-Fermentation Properties of Dough

**Figure 2:**
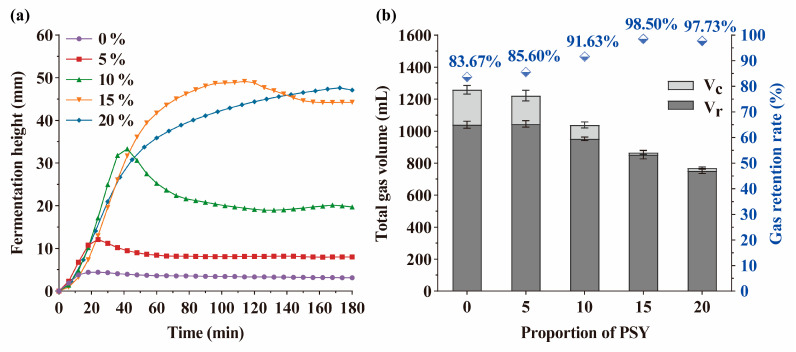
The dependence of rheo-fermentation properties of dough on the proportions of PSY. (**a**) Different proportions of PSY (0, 5, 10, 15, and 20%) were included in the dough, and the fermentation height of the dough was determined. (**b**) The effects of PSY on the gas volume and retention rate of dough were determined. V_c_, loss gas volume; V_r_, retention gas volume. The experiments were performed in triplicate.

The fermentation process of the dough plays a crucial role in determining the quality of bread [38]. The expansion capacity of the dough during fermentation is typically influenced by the dough structure and the amount of gas retained [33]. Here, although the control dough exhibited the highest retention gas volume, the maximum fermentation height was only 4.5 mm during fermentation, and the fermentation height of the dough was increased with the addition of PSY (Figure 2a). Notably, the maximum fermentation height was significantly increased to 49.9 mm when the amount of PSY was raised to 15%. This increased fermentation height remained stable throughout the prolonged fermentation time. The retention rate of gas also reached a maximum of 98.5% with the inclusion of 15% PSY (Figure 2b). Taken together, these results suggest that the addition of PSY leads to a higher fermentation height because it improves the gas retention rates of dough during the proving process. The decrease in total gas volume observed with the addition of PSY can be attributed to the reduction in the content of buckwheat flour in the dough. 

### 3.3. Microscopic Analysis of Dough Properties

**Figure 3:**
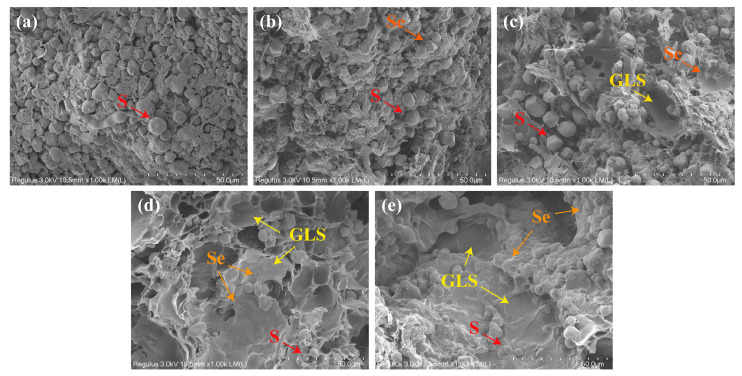
Dependence of dough microstructure on the proportion of PSY. (**a**–**e**) SEM images of dough samples. S, starch particles; Se, starch particles embedded in the dough; GLS, gluten-like structure.

A microstructural analysis was conducted to further probe the effects of PSY addition on dough properties. As shown in Figure 3a, in the control dough, starch granules were predominantly exposed, and no continuous and uniform matrix was observed. This lack of a continuous structure may be an important factor contributing to the poor gas-holding capacity in the control dough. The addition of 5% PSY resulted in the covering of some of the surfaces of the starch granules, which correlated with an enhanced stability of the dough matrix (Figure 3b). When the amount of PSY reached 10%, a gluten-like structure (GLS) emerged, with more starch granules embedded within the dough matrix (Figure 3c). Consistent with these findings, previous studies have also demonstrated that hydrocolloids such as hydroxypropyl methylcellulose and xanthan gum can cover the surfaces of starch particles and form GLS in GF doughs [33,39]. Notably, the dough that contained 15% PSY exhibited a more continuous and compact GLS structure (Figure 3d). We suggest that the GLS in dough that included PSY was an important factor in its enhanced gas-holding capacity and increased volume. 

### 3.4. Analysis of Specific Volume and Baking Loss of Bread

**Figure 4:**
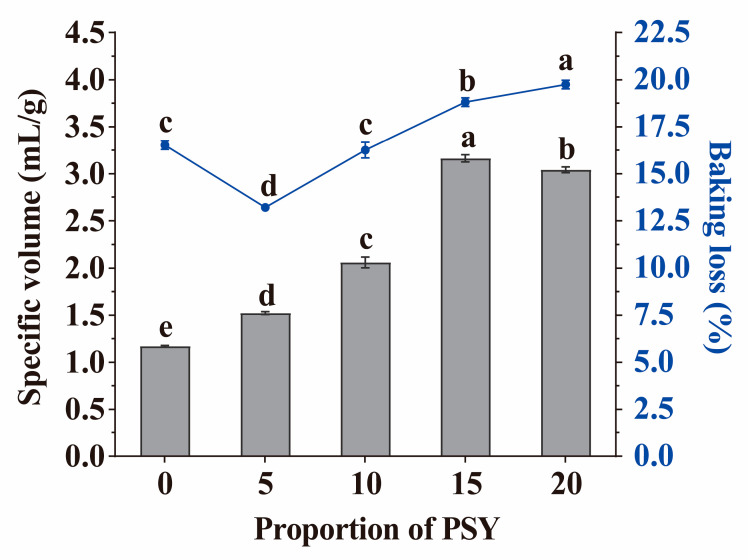
The specific volume and baking loss of bread with different proportions of PSY. Letters indicate significant differences (*p* < 0.05). The experiments were performed in triplicate.

The specific volume of GF bread is another factor that influences its quality and directly affects consumer preferences [26]. As depicted in Figure 4, the addition of PSY from 0% to 15% in GF bread led to a significant increase in specific volume, from 1.17 mL/g to 3.16 mL/g. When the amount of PSY increased from 15% to 20%, a slight reduction in specific volume was observed. During the baking process, a reduction in the weight of bread primarily occurs due to water evaporation. Compared with the control dough, the addition of 5% PSY resulted in a decrease in baking loss from 16.52% to 13.20%. This reduction can be attributed to the strong water-holding capacity of PSY. However, when the amount of PSY exceeded 5%, the baking loss increased as the amounts of PSY increased. This change was attributed to the higher water content in the dough that included PSY and the formation of a porous structure due to the addition of PSY [19].

### 3.5. The Physical Appearance of Bread

**Figure 5:**
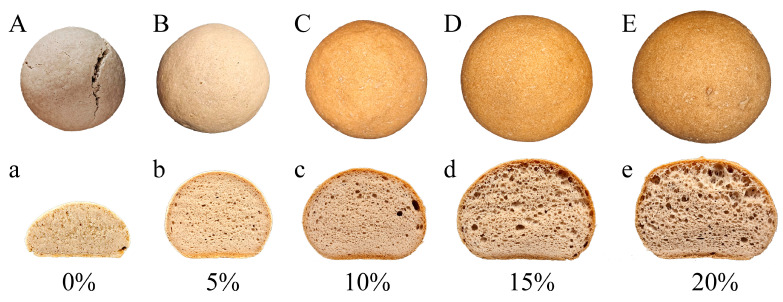
The dependence of the physical appearance of bread crust and crumb on the proportions of psyllium fibre (PSY). (**A**–**E**) Representative images of bread crusts. (**a**–**e**) Representative images of bread crumbs. Images are representative of at least three independently produced batches of bread.

The colour of bread is a significant factor in its appeal to consumers; GF bread often has a lighter hue that is less appealing to consumers. As illustrated in Table 1, as the proportion of PSY increased, the L* value, which is an indicator of the darkness of the crust, decreased and the a* and b* values, which indicate red and green or blue and yellow hues, increased. The crust of PSY-added bread displayed a toast tan colour (Figure 5), which can be attributed to the enhancement in the Maillard reaction during baking [40]. Meanwhile, the L*, a*, and b* values of the bread crumbs also increased with the addition of PSY to the dough, primarily due to the natural colouring properties of PSY itself. As is shown in Figure 5A, the control bread exhibited cracks on its crust owing to the low water-holding capacity of the dough. The addition of PSY resulted in bread with improved physical characteristics, such as even crusts and an absence of cracks (Figure 5b–e). The addition of PSY enhanced the overall appearance and visual appeal of the bread.

### 3.6. Textural and Structural Properties of Bread

**Figure 6:**
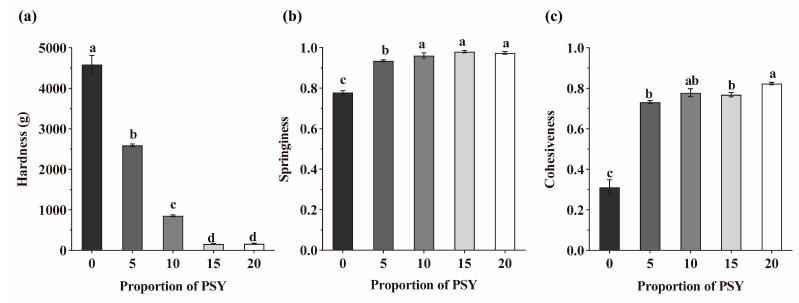
Textural properties of bread with different proportions of PSY. (**a**–**c**) The hardness (**a**), springiness (**b**), and cohesiveness (**c**) values of breads made with the noted proportions of PSY were determined with a texture analyser. Letters indicate significant differences (*p* < 0.05).

The textural properties of bread crumbs, particularly their hardness and springiness, have been shown to have an important impact on bread quality [41]. In addition, previous studies have indicated a negative correlation between bread hardness and specific volume [39]. As shown in Figure 6a, we observed that the control bread had the highest level of hardness and the smallest specific volume. The addition of PSY reduced the hardness of the bread crumb, with notable improvements observed at 15% and 20% PSY additions. This reduction in hardness can be attributed to the increased bread volume and the presence of more pores when PSY was added to the dough. It should be noted that at a PSY addition of 20%, larger pores were formed, negatively impacting bread quality. This is due to the high moisture content upon the addition of higher proportions of PSY, which weakens the gas cell walls and promotes the aggregation of small pores [42].

Springiness is generally positively correlated with sensory properties [43]. The addition of 15% PSY increased the springiness of the bread from 77.87% to 98.03%, indicating that the addition of PSY facilitated the formation of a network structure, thereby improving the bread’s resistance to deformation (Figure 6b). Furthermore, the addition of PSY also improved bread cohesiveness, resulting in a more solid and less crumbly bread matrix (Figure 6c). It is worth noting that similar improvements in cohesiveness of GF breads have been observed with the addition of xanthan gum and apple pectin [39].

### 3.7. In Vitro Analyses of Starch Digestibility 

**Figure 7:**
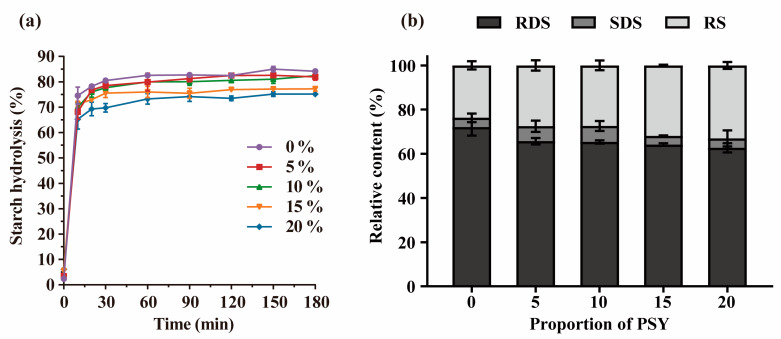
The starch hydrolysis curve (**a**) and starch compositions (**b**) of bread with different proportions of PSY. RDS, rapidly digestible starch; SDS, slowly digestible starch; and RS, resistant starch.

Figure 7 illustrates the impact of PSY on the starch digestion of the GF bread. The samples underwent a rapid hydrolysis process within the first 10 min when exposed to a simulated digestive fluid. Subsequently, the rate of starch hydrolysis slowed down and gradually reached equilibrium after 60 min of digestion (Figure 7a). These results indicate that the inclusion of PSY in GF-free bread can effectively modulate the rates of starch digestion, resulting in a slower release of glucose. To evaluate the effect of PSY on the digestion of starch from GF bread, a first-order kinetic model was used to fit the hydrolysis curve [44]. The equilibrium amount of starch digested (*C_∞_*) decreased from 82.93% to 75.09% as the level of PSY increased from 0% to 20% (Table 2). This reduction can be attributed to the formation of a protective layer by PSY on the starch surface, acting as a physical barrier that impedes starch digestion (Figure 3). 

**Figure 8:**
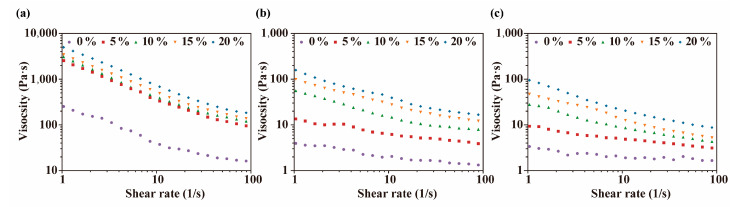
Rheological analyses of the digesta of breads with varying proportions of PSY. (**a**–**c**) Steady shear viscosity of the digesta at three different digestion times: 0, 20, and 120 min, respectively.

Compared with the digesta without PSY, the digesta containing PSY exhibited higher viscosity (Figure 8). The elevated viscosity associated with PSY-containing digesta can potentially hinder fluidity, restricting the interaction between enzymes and substrates, consequently resulting in the observed decrease in starch digestibility [45]. The reduction in digesta viscosity during the digestion process was attributed to the decomposition of bread by digestive enzymes, which leads to a reduction in particle size, as well as the disruption of the network structure formed by PSY and starch [46]. Additionally, the starch digestion rate coefficient (*k*) did not show a significant decline with the addition of PSY. 

Figure 7b presents the distribution of starch fractions in GF bread as a function of PSY addition. Starch can be categorised into three groups based on its rate and extent of starch digestion: rapidly digestible starch (RDS), slowly digestible starch (SDS), and resistant starch (RS) [47]. With the addition of 15% PSY, there was a decrease in RDS content from 72.03% to 64.19% and an increase in RS content from 23.72% to 31.96% when compared with the control bread (Table 3). Lower RDS levels and higher RS levels have previously been linked to reduced glycaemic index (GI) values [48,49]. These findings suggest that the inclusion of PSY in GF bread leads to an altered starch composition, with a decrease in RDS content and an increase in RS content. This change in starch fractions would be expected to contribute to a lower GI value for the bread, indicating a potential positive effect of PSY on glycaemic control.

## 4. Conclusions

To address the technological challenges in the production of GF bread and enhance its structural acceptability, our research demonstrates the potential of using PSY as an ingredient to produce high-quality GF bread with a lower glycaemic index. Our findings indicate that the inclusion of PSY results in the development of a continuous and dense network structure in the dough, leading to GF bread with superior characteristics such as increased volume and reduced hardness. Additionally, the incorporation of PSY contributes to the desirable appearance of the bread, as evidenced by a toasted tan colour observed on the crust, making it more visually appealing and aesthetically pleasing. The addition of PSY also has a positive impact on the nutritional composition of GF bread. It helps to lower blood glucose levels by reducing the digestibility of starch. Moreover, PSY increases the amount of RS, which has notable benefits for digestion and has the potential to improve gut health.

The rising demand for superior quality GF bread, as well as the growing consumer preference for natural products, requires the exploration of innovative approaches in GF breadmaking. Our findings suggested that incorporating PSY into GF bread has the potential to meet the increasing demand for high-quality, healthier GF options by enhancing both the texture and nutritional profile of the bread. 

## Figures and Tables

**Table 1:** Dependence of colour properties of bread on the proportion of PSY.

PSY Proportion		Crust			Crumb	
	L*	a*	b*	L*	a*	b*
0%	67.81 ± 0.92 ^a^	0.24 ± 0.03 ^d^	15.34 ± 0.37 ^c^	46.70 ± 0.95 ^c^	1.13 ± 0.10 ^d^	8.79 ± 0.16 ^b^
5%	58.03 ± 0.50 ^b^	3.12 ± 0.05 ^c^	15.40 ± 0.03 ^c^	47.78 ± 0.93 ^c^	1.72 ± 0.15 ^c^	9.22 ± 0.15 ^b^
10%	49.86 ± 0.93 ^c^	5.21 ± 0.04 ^b^	17.52 ± 0.20 ^a^	51.40 ± 0.68 ^b^	2.32 ± 0.13 ^b^	9.77 ± 0.38 ^ab^
15%	46.55 ± 0.17 ^d^	5.64 ± 0.26 ^a^	17.13 ± 0.31 ^ab^	54.33 ± 0.58 ^a^	2.51 ± 0.05 ^b^	9.78 ± 0.18 ^ab^
20%	45.83 ± 0.23 ^d^	5.92 ± 0.10 ^a^	16.77 ± 0.16 ^b^	53.95 ± 0.34 ^a^	2.95 ± 0.09 ^a^	9.99 ± 0.24 ^a^

L*: light/dark; a*: red/green shade; and b*: yellow/blue shade. Letters indicate statistical significance (*p* < 0.05) within a column.

**Table 2:** The in vitro kinetics of digestion of starch from bread prepared with different proportions of PSY.

PSY Proportion	*C_∞_* (%)	*k* (min^−1^)	R^2^
0%	82.93	0.1533	0.9981
5%	82.11	0.1391	0.9942
10%	80.26	0.1982	0.9802
15%	76.45	0.1973	0.9867
20%	75.09	0.1560	0.9893

*C_∞_:* the equilibrium amount of starch digested; *k*: first-order kinetic coefficient.

**Table 3:** The effects of PSY on the starch compositions of bread.

PSY Proportion	RDS (%)	SDS (%)	RS (%)
0%	72.03 ± 3.80 ^a^	4.25 ± 1.92 ^a^	23.72 ± 1.88 ^b^
5%	65.70 ± 1.42 ^b^	6.79 ± 2.56 ^a^	27.51 ± 2.33 ^b^
10%	65.35 ± 0.71 ^b^	7.24 ± 2.25 ^a^	27.41 ± 2.20 ^b^
15%	64.19 ± 0.61 ^b^	3.85 ± 0.31 ^a^	31.96 ± 0.32 ^ab^
20%	62.71 ± 2.05 ^b^	4.25 ± 3.62 ^a^	33.04 ± 1.57 ^a^

RDS, rapidly digestible starch; SDS, slowly digestible starch; and RS, resistant starch. Letters indicate significant differences (*p* < 0.05) within a column.

## Data Availability

The original contributions presented in the study are included in the article, further inquiries can be directed to the corresponding author.
